# T1 Burn Behavioral Health: Development and Preliminary Results of Burn Center-Based Stepped-Care Program for Burn Survivors

**DOI:** 10.1093/jbcr/irad045.001

**Published:** 2023-05-15

**Authors:** Yulia Gavrilova, Raleigh Cerre, Julia Ficalora, Davidson Tatiana, Kenneth Ruggiero, Aaron Lesher, Steven A Kahn

**Affiliations:** The South Carolina Burn Center / Medical University of South Carolina, Charleston, South Carolina; Medical University of South Carolina, Charleston, South Carolina; Medical University of South Carolina, Charleston, South Carolina; Medical University of South Carolina, Charleston, South Carolina; Medical University of South Carolina, Charleston, South Carolina; South Carolina Burn Center, Charleston, South Carolina; The South Carolina Burn Center/Medical University of South Carolina, Charleston, South Carolina; The South Carolina Burn Center / Medical University of South Carolina, Charleston, South Carolina; Medical University of South Carolina, Charleston, South Carolina; Medical University of South Carolina, Charleston, South Carolina; Medical University of South Carolina, Charleston, South Carolina; Medical University of South Carolina, Charleston, South Carolina; South Carolina Burn Center, Charleston, South Carolina; The South Carolina Burn Center/Medical University of South Carolina, Charleston, South Carolina; The South Carolina Burn Center / Medical University of South Carolina, Charleston, South Carolina; Medical University of South Carolina, Charleston, South Carolina; Medical University of South Carolina, Charleston, South Carolina; Medical University of South Carolina, Charleston, South Carolina; Medical University of South Carolina, Charleston, South Carolina; South Carolina Burn Center, Charleston, South Carolina; The South Carolina Burn Center/Medical University of South Carolina, Charleston, South Carolina; The South Carolina Burn Center / Medical University of South Carolina, Charleston, South Carolina; Medical University of South Carolina, Charleston, South Carolina; Medical University of South Carolina, Charleston, South Carolina; Medical University of South Carolina, Charleston, South Carolina; Medical University of South Carolina, Charleston, South Carolina; South Carolina Burn Center, Charleston, South Carolina; The South Carolina Burn Center/Medical University of South Carolina, Charleston, South Carolina; The South Carolina Burn Center / Medical University of South Carolina, Charleston, South Carolina; Medical University of South Carolina, Charleston, South Carolina; Medical University of South Carolina, Charleston, South Carolina; Medical University of South Carolina, Charleston, South Carolina; Medical University of South Carolina, Charleston, South Carolina; South Carolina Burn Center, Charleston, South Carolina; The South Carolina Burn Center/Medical University of South Carolina, Charleston, South Carolina; The South Carolina Burn Center / Medical University of South Carolina, Charleston, South Carolina; Medical University of South Carolina, Charleston, South Carolina; Medical University of South Carolina, Charleston, South Carolina; Medical University of South Carolina, Charleston, South Carolina; Medical University of South Carolina, Charleston, South Carolina; South Carolina Burn Center, Charleston, South Carolina; The South Carolina Burn Center/Medical University of South Carolina, Charleston, South Carolina

## Abstract

**Introduction:**

Annually, nearly half a million people in the U.S. receive medical treatment for burns, 40,000 of whom are hospitalized. Burn patients experience significant psychological burden during acute burn treatment. Long-term psychiatric problems are highly prevalent compared to patients with other injuries. Undetected or untreated psychiatric symptoms can complicate recovery, increase length of stay, and cause long-term problems and readmission. Burn centers are ideally suited to provide these services to burn patients on an inpatient and outpatient basis; however, few burn centers in the U.S. have mental health programs to address patients’ needs. This presentation will describe the *Burn Behavioral Health (BBH)* program, a burn center-based technology-enhanced stepped-care model of delivering mental health services for burn survivors, spanning the inpatient to outpatient continuum. This model was adapted from the Trauma Resilience and Recovery Program which has been successful in meeting the needs of traumatic injury patients.

**Methods:**

Data were collected as part of clinical care. *BBH* includes 4 main steps: (1) initial screening, education, and early intervention; (2) symptom self-monitoring and self-help resources; (3) follow-up screening 30 days post-injury; and (4) provision of best-practice mental health treatment for patients who need it, using both in-person and telehealth modalities, including individual and skill-based group therapy.

**Results:**

Between February 2021 and September 2022, 384 patients were identified as eligible for *BBH* services. Of these, 342 (90%) were enrolled in *BBH* and completed initial screening. 50% of patients screened positive for depression/PTSD risk or symptoms and were offered brief intervention. 54% of enrolled survivors received brief intervention, 21% of whom completed more than one session. We reached 84% of patients for the 30-day follow-up, 20% of whom screened positive for depression/PTSD and 29% were connected to mental health services.

**Conclusions:**

This study provides preliminary support demonstrating that *BBH* is sustainable and capable of reaching and engaging a large proportion of burn patients across the inpatient to outpatient continuum. In the U.S., only 25% of burn centers have structured screening in place for inpatient and 11% for outpatient burn survivors. Data from the traumatic injury population (which includes burns) suggest only 10% of patients received intervention after a positive screen and 42% did not know how or where to get help. Psychological recovery is an essential component of a comprehensive burn program and availability of psychological screening and intervention is a requirement for the ABA verification.

**Applicability of Research to Practice:**

*BBH* shows promise in increasing access and quality of mental health care to address the unique needs of burn survivors.

